# Ear diseases among secondary school students in Xi'an, China: The role of portable audio device use, insomnia and academic stress

**DOI:** 10.1186/1471-2458-11-445

**Published:** 2011-06-08

**Authors:** Yang Chen, Xu Li, Zhan Xu, Zonghua Li, Pengzhi Zhang, Ya He, Fangyuan Wang, Jianhua Qiu

**Affiliations:** 1 Department of Otolaryngology-Head and Neck Surgery, Xijing Hospital, Fourth Military Medical University, Xi'an-710032, China

## Abstract

**Background:**

Hearing impairment negatively impacts students' development of academic, language and social skills. Even minimal unilateral hearing loss can hinder educational performance. We investigated the prevalence of ear diseases among secondary school students in the city of Xi'an, China in order to provide a foundation for evidence-based hearing healthcare.

**Methods:**

A stratified random sampling survey was conducted in 29 secondary schools. Demographics and medical histories were collected, and otologic examinations were performed. Questionnaires were administered to assess insomnia, academic stress and use of portable audio devices. Logistic regression analysis was used to identify factors associated with hearing impairment, and the association of sensorineural hearing loss with insomnia, academic stress and the use of portable audio devices was analyzed with the chi-square test.

**Results:**

The percentage of students with some form of ear disease was 3.32%. External ear disease, middle ear disease and sensorineural hearing loss occurred in 1.21%, 0.64% and 1.47% of the students, respectively. Boys had a relatively higher prevalence of ear disease than girls. According to our survey, the prevalence of sensorineural hearing loss increased significantly among the students with insomnia and extended use of portable audio devices, but not among those with elevated levels of academic stress. Hearing aids and surgical treatment were needed in 1.47% and 0.89% of the students, respectively.

**Conclusions:**

There is a high prevalence of ear disease among secondary school students, and this should be given more attention. Insomnia and the excessive use of portable audio devices may be related to adolescent sensorineural hearing loss. It is important to establish and comply with an evidence-based preventive strategy.

## Background

Ear diseases may cause life-long or, sometimes, life-threatening problems. Congenital or acquired hearing impairment (HI) hinders students' development of academic, language and social skills. Even minimal unilateral sensorineural hearing loss (SHL) can have a negative impact [1-4], and bilateral SHL can result in psychological problems. The World Health Organization (WHO) estimated that 278 million people in the world, two-thirds of whom were in developing countries, suffered from moderate to profound HI in 2005. It was also estimated that at least 68 million people have had HI since childhood [5]. These problems produce surprisingly large economic burdens on society as a whole. Therefore, early otologic diagnosis and intervention are important [6].

Due to the impact of HI on mental and physical health, the WHO formulated the Ear and Hearing Disorders Survey Protocol in 1999. While hearing screening is routinely carried out among school-age children in developed countries, in some developing countries, there is not a consummate procedure to prevent or treat ear diseases promptly and effectively. In China, a developing country with the highest population in the world, hearing disabilities have been one of the most common types of disabilities. The second national disability survey in 2006 reported a hearing disabled population of 27.8 million in China. In two investigations conducted in eastern China, 10% of 442 urban students and 20% of 282 rural students, ranging from 6 to 19 years of age, were diagnosed as HI [7,8].

A recent study showed that portable audio device-induced hearing loss was evolving into a significant social and public health problem [9]. HI contributes not only to academic difficulty but also to sleep impairment, especially insomnia [10]. Therefore, it is important to explore the association of SHL with study stress, insomnia and the use of portable audio devices. In this study, we determined the prevalence of ear disease among secondary school students in Xi'an, a provincial capital city in western China. Furthermore, related social behaviors and their associations with SHL were investigated and are discussed below.

## Methods

### Participants

This cross sectional study, conducted between April 2009 and July 2009, investigated the distribution of ear diseases among secondary school students in Xi'an. Particular attention was paid to the prevalence and characteristics associated with SHL. Students from 29 secondary schools in Xi'an were selected using a stratified sampling method. Ten schools were randomly selected from the 29 schools, and three grades of each school were selected according to a random table. The total number of participants was 1567 (541, 557 and 469 in grades eight, ten, and eleven, respectively).

This study was approved by the Institutional Review Board of the Ethics Committee of Xijing Hospital, the Fourth Military Medical University of the People's Liberation Army of China. Written informed consents were obtained from all families and participants.

### Otoscopy

Each ear was examined by pneumatic otoscopy. All abnormal findings were confirmed by a trained otolaryngologist.

### Audiometry

The pure tone test was performed in a sound-proof audiometric room in Xijing Hospital (OB922 pure tone audiometer, Madsen, Denmark, bio-calibrated daily). Air conduction was performed for 250, 500, 1000, 2000, 4000, and 8000 Hz. Bone conduction was performed for 250, 500, 1000, 2000, and 4000 Hz. All tests followed the Hughson-Westlake ascending method. Narrow band noise masking was applied to the opposite ear when the difference in threshold was no less than either 40 dB between the air conduction of the test and non-test ear or 15 dB between the air and bone conduction of the test ear.

### Tympanometry

Tympanometry was performed with the acoustic impedance instrument ZODIAC901 (Madsen, Denmark). Tympanic compliance, middle ear pressure, and tympanogram were recorded.

### Questionnaire

A survey detailing each student's demographics, medical history, and noise exposure was administered. With respect to noise exposure, the average use time and volume of portable audio devices was recorded. Two validated questionnaires, the Chinese Mental Health Inventory for Middle School Students (CMHI) [11] and the Athens insomnia scale (AIS) [12], were conducted to reflect academic stress and insomnia.

Sleep (reflected by AIS) was defined as normal (0-3 points), probable sleeping disorder (4-5 points), or insomnia (≥6 points).

According to the exposure level, portable audio device use was categorized as control (without a portable audio device), low exposure (less than 0.5 hour per day), medium exposure (0.5-1.5 hours per day), and high exposure (more than 1.5 hours per day). "Loud sound" (≥85 dB) was defined as frequently using more than two-thirds of the maximum volume.

### Diagnostic criteria

According to the WHO/International Organization for Standardization (ISO) definition, participants were diagnosed as HI when the average threshold (500, 1000, 2000, and 4000 Hz) was more than 25 decibels of hearing loss (dB HL) in either ear. The severity of HI (based on the pure tone average in the better ear) was categorized as mild (26-40 dB HL), moderate (41-60 dB HL), severe (61-80 dB HL), or profound (> 80 dB HL). HI was further classified as conductive, sensorineural, or mixed.

Before investigating the association between SHL and portable audio device use, we excluded the subjects with middle ear disease, congenital deafness, drug-induced deafness, history of head injury, familial deafness, or other ear organic disease.

### Statistical analysis

The data were entered into EpiData 3.0 software and verified by two of the authors. The chi-square test was performed to show the association of SHL with related behaviors. All variables (gender, age, portable audio device use, academic stress, sleep disorder, history of ototoxic drug administration and family history of deafness) were included in a logistic regression model to identify correlation factors for HI. Odds ratios and 95% confidence intervals were calculated (SPSS version 15.0; SPSS Inc., Chicago, Ill.). A P-value of less than 0.05 was considered statistically significant.

## Results

### Percentage of identified ear diseases

A total of 1567 students (793 boys, 774 girls) were enrolled between April 2009 and July 2009. The ages ranged from 12 to 19 with a mean of 15.7 years. Following the WHO definitions, 23 of 1567 participants had mild HI, 4 had moderate HI, 2 had severe HI, and 1 had profound HI (Table 1). Among the students with impaired hearing, 4 had conductive hearing impairment loss (23.33%), 3 had mixed hearing loss (10%), and 23 had SHL (66.67%). Bilateral hearing loss was detected in 19 of the 30 students with HI.

**Table 1:** HI of different level

Ear diseases	Number	Percentage(%)
*Mild (26 - 4 dB)*	23	1.47
External auditory canal stenosis	1	
Active stage of chronic suppurative otitis media	3	
Secretory otitis media	1	
Tympanic membrane perforation (dry ear)	1	
Tympanosclerosis	1	
Hereditary	3	
Noise-induced	12	
Unknown	1	
*Moderate (41 - 60 dB)*	4	0.26
Tympanosclerosis	1	
Hereditary	1	
Noise-induced	2	
*Severe (61 - 80 dB)*	2	0.13
Drug-induced	1	
Unknown	1	
*Profound (> 80 dB)*	1	0.06
Unknown	1	

The percentage of students with ear disease was 3.32% in total. External ear disease, middle ear disease and SHL accounted for 1.21%, 0.64%, and 1.47%, respectively (Table 2). As shown in Table 3 the percentage of ear disease among boys (5.30%) was significantly higher than that among girls (1.29%, *P *< 0.001). The risk for the former was 4.273-fold higher than the latter (95% confidence interval 2.128-8.578). There was no significant difference in the percentage of ear disease among different ages.

**Table 2:** Percentage of identified ear diseases

Ear diseases	Number	Percentage(%)
*External ear diseases*	19	1.21
Accessory auricle	7	0.45
Congential preauricular fistula	8	0.51
External auditory canal stenosis	2	0.13
Otisis externa	1	0.06
First branchial cleft fistula	1	0.06
*Middle ear disease*	10	0.64
Active stage of chronic supprative otisis media	3	0.19
Secretory otitis media	2	0.13
Tympanic membrane perforation(dry ear)	3	0.19
Tympanosclerosis	2	0.13
*Sensorineural hearing loss*	23	1.47
Hereditary	4	0.26
Drug-induced	1	0.06
Noise-induced	14	0.89
Unknown	4	0.26

**Table 3:** Percentage of ear disease stratified by gender and age

Gender and Age		Ear disease	Without ear disease	Total	Percentage of ear disease (%)
Gender^1^	Boy	42	751	793	5.30%
	Girl	10	764	774	1.29%
Age^2^	12-14	13	408	421	3.09%
	15-17	36	993	1029	3.50%
	18-19	3	114	117	2.56%

*X^2^_1 _*= 19.576, *P *= 0.000; *X^2^_2 _*= 0.381, *P *= 0.826

In a multiple logistic regression model (Table 4), gender, use of portable audio devices, sleep disorders, history of ototoxic drug administration and family history of deafness were associated with HI, while age was not. In addition, use of portable audio devices was an independent risk factor of HI. Female gender was a protective factor for HI.

**Table 4:** Multiple logistic regression analysis of HI

Variable	*OR *value	95% *CI*	*P *value
Gender	0.342	0.250-0.638	0.004
Age	1.350	0.555-3.284	0.532
Portable audio device use	8.676	5.176-12.462	0.026
Academic stress	5.006	0.984-11.280	0.095
Sleep disorder/insomnia	1.925	1.040-4.377	0.035
History of ototoxic drug administration	2.316	1.071-5.258	0.046
Family history of deafness	3.518	1.124-4.334	0.009

*OR *(odds ratio), *CI *(confidence interval)

### SHL-related social behaviors

SHL was not associated with academic stress scores (χ^2 ^= 4.065, P = 0.255) (Table 5). However, it was significantly increased among those students with a sleeping disorder and insomnia (χ^2 ^= 9.761, P = 0.008) (Table 6). By logistic regression analysis, we found a greater risk of SHL when the insomnia score was more than 6 (P = 0.002, α = 0.015).

**Table 5:** Percentage of SHL stratified by academic stress level

Academic stress	SHL	Without SHL	Total	Percentage of SHL(%)
Normal	14	922	936	1.50
Mild	6	514	520	1.15
Moderate	2	96	98	2.04
Severe	1	12	13	7.69
	
Total	23	1544	1567	1.47

*X^2 ^*= 4.065, *P *= 0.255

**Table 6:** Percentage of SHL stratified by sleep problems

AIS Score	SHL	Without SHL	Total	Percentage of SHL(%)
Normal	19	1282	1301	1.46
Sleeping disorder	3	257	260	1.15
Insomnia	1	5	6	16.67
	
Total	23	1544	1567	1.47

*X^2 ^*= 9.761, *P *= 0.008

**Figure 1:**
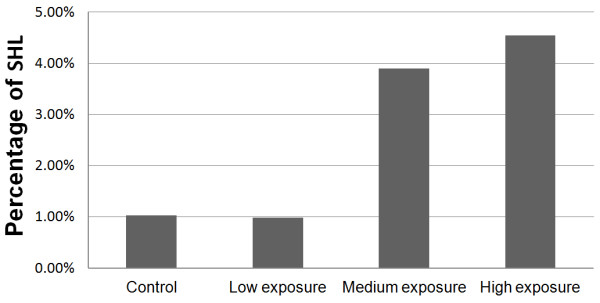
**Percentage of SHL among students who use portable audio devices**. The percentage of students with SHL in control, low exposure, medium exposure and high exposure strata were 1.04%, 0.99%, 3.91% and 4.55%, respectively.

According to our survey, 1335 (85.19%) students used portable audio devices. Among these subjects, 1098 (82.20%) students preferred loud sound (≥85 dB). The average use time was 1.41 ± 1.11 hours per day. SHL was associated with the extent of portable audio device use (*χ^2 ^*= 8.15, *P *= 0.043) and they were positively correlated (Figure 1).

### Intervention needed

According to the degree of HI, different interventions were advised: mild HI warranted a consultation and consideration of a hearing aid; for moderate HI a hearing aid was recommended; severe HI warranted a hearing aid, lip reading, and sign language instruction; and profound HI warranted a hearing aid, rehabilitation, lip reading, and sign language instruction. In total, 80 participants (5.11%) needed medical interventions. Hearing aids were needed by 23 students (1.47%). Non-emergency ear surgeries were required for 14 students (0.89%) (10 cases of middle ear disease, 2 cases of congenital preauricular fistula with infection history and 2 cases of external auditory canal stenosis). Additionally, 29 boys (3.66%) and 13 girls (1.68%) had cerumen impaction, and they all needed cerumen removal. One student with otitis externa needed medical treatment. One student with profound SHL needed speech rehabilitation.

## Discussion

This is the first study investigating the prevalence of ear diseases among Chinese adolescents using a stratified sampling method. Although it is only a local and partial survey, the current situation of ear diseases among urban secondary school students in Northwest China can be extrapolated. This survey provides scientific evidence for China and other developing countries to develop adolescent HI prevention and control strategies.

The observation that 3.32% percent of students have some form of ear disease suggests an urgent need for otologic health care in adolescents. Many of the cases affected the external ear, including the accessory auricle, the congenital preauricular fistula, the external auditory canal stenosis, the otitis externa and the first branchial cleft fistula. Although external ear diseases usually do not cause direct hearing and balancing damage, they may place a child at risk for developing damage. Middle ear disease was represented mainly by chronic purulent otitis media and secretory otitis media. The small proportion (19.23%, 10/52) of middle ear diseases is probably ascribed to the extensive application of antibiotics and the decreased incidence of secretory otitis media with physical development. However, the excessive treatment of secretory otitis media may lead to tympanic sclerosis during childhood [13], and, in fact, we observed two cases of tympanic sclerosis in our study. Therefore, the long-term effects of treatment should be carefully considered when making a surgical decision. The low proportion of chronic purulent inflammation we observed was consistent with the survey by Zakzouk *et al*. [14], which showed a decrease in urban incidence of chronic suppurative otitis media (CSOM) over the past two decades. Nevertheless, we need to remain vigilant because of the severe extracranial and intracranial complications due to chronic purulent inflammation. The majority of people were not aware of these ear diseases. Therefore, it is important to put some effort into otologic health education among school students.

In this survey, the most common ear disease (44.23%, 23/52) was SHL caused by noise, drugs, heredity, CSOM, otosclerosis and unidentified factors. SHL can cause life-long and sometimes life-threatening difficulties and it can profoundly affect the ability to communicate as well as hinder education and employment. Therefore, education on ear health deserves more attention in schools.

Congenital ear disease accounts for 42.31% (22/52) of the ear diseases we observed. It causes concern not only because of the possible damage to hearing or balance, but also due to its heritability and association with hereditary diseases in other organs.

According to our survey, the percentage of ear disease among boys was significantly higher than that among girls. While this is consistent with a study from the Jiangsu Province of China [7], it is contrary to that of Shargorodsky, et al. in 2010 [15]. The discrepancy may result from differences in the genetic backgrounds of the study populations or the increased difference in use of portable audio and gaming devices by boys compared to girls in China. HI didn't correlate with age in our study, which may be a result of the minimal age difference of the subjects.

The percentage of students with SHL did not differ among the students with elevated levels of academic stress. However, because 40.27% of the students reported experiencing at least mildly elevated levels of academic stress, schools and communities may want to address this issue. The percentage of SHL increased significantly among those students with insomnia. Because there is not enough evidence to decide the exact causality, further studies are necessary to determine the relationship between insomnia and SHL.

Among all causes of SHL, noise was the most common (60.87%, 12/23). With the economic development of China, industrial, transportation-related and entertainment-related noise pollution has become increasingly prominent. While a temporary threshold shift in noise induced hearing loss is reversible, it is difficult to stop the progression of the threshold shift from temporary to permanent. It was recently reported that 3.4% of female and 10.3% of male youths in the US used hearing-protection in 2005-2006 [16]. However, the students in our study lacked awareness of hearing protection principles. Therefore, an important method for reducing HI is to provide hearing health care education and guide adolescents to use portable audio and gaming devices appropriately.

This study only investigated ear diseases among urban and suburban students, not rural students. Studies covering a more extensive and varied area are needed. In addition, otologic eikonic data, important for the etiological diagnosis of SHL, were not collected.

This study suggests that the education and popularization of hearing health knowledge among adolescents should be prioritized. Otologists should reinforce the prevention and rehabilitation of HI among young people while technical services and the appropriate help should be provided to people with hearing and speech disabilities. Cerumen impaction accounted for a significant proportion of ear problems. Although cerumen removal should be carried out in a hospital, non-medical cerumen removal was very common. This practice, and the excessive use of portable audio devices, are two examples of bad habits those should be corrected to avoid the potential risk of HI.

## Conclusions

More attention should be paid to the high prevalence of ear disease among secondary school students. Insomnia and the excessive use of portable audio devices may be associated with adolescent SHL. It is important to establish and comply with an evidence-based preventive strategy.

## Competing interests

The authors declare that they have no competing interests.

## Authors' contributions

JQ initiated and designed the survey. YC was responsible for the organization of the survey and the division of labor. XL was in charge of data collection and manuscript drafting. ZX, ZL, PZ, YH and FW performed the otologic examinations and administered the questionnaires. All authors read and approved the final manuscript. JQ is guarantor of the paper.

## Pre-publication history

The pre-publication history for this paper can be accessed here:

http://www.biomedcentral.com/1471-2458/11/445/prepub
